# Modulation of cGMP/cAMP crosstalk on the level of PDE3 in animals lacking NO-sensitive guanylyl cyclase

**DOI:** 10.1186/2050-6511-16-S1-A52

**Published:** 2015-09-02

**Authors:** Andreas Friebe, Sarah Dünnes, Viacheslav Nikolaev, Barbara Lies, Dieter Groneberg

**Affiliations:** 1 Institute of Physiology, University of Würzburg, Würzburg, Germany; 2 Institute of Experimental Cardiovascular Research, University Medical Center Hamburg-Eppendorf (UKE), Hamburg, Germany

## 

NO-sensitive guanylyl cyclase (NO-GC) is accepted to be the major receptor for the signaling molecule NO. Deletion of NO-GC in mice leads to disturbed NO/cGMP signaling. As a result, these mice show abolished NO-dependent relaxation of smooth muscle-containing tissues in the cardiovascular and gastrointestinal systems. Mice with general deletion (GCKO) suffer from increased blood pressure, reduced bleeding time, erectile dysfunction and die prematurely due to gastrointestinal dysmotility. Mice lacking NO-GC specifically in smooth muscle cells (SMC) show an elevated blood pressure similar to that seen in GCKO animals.

Crosstalk between cGMP and cAMP signaling has been reported to occur in many different cells. Phosphodiesterase 3 (PDE3) is thought to be one of the prominent mediators of this crosstalk. Through its inhibitory action on PDE3, cGMP may regulate cAMP levels and cAMP-mediated signaling. Here, we reasoned that, in animals deficient in NO-GC, lack of NO-induced cGMP synthesis might affect cGMP/cAMP crosstalk on the level of PDE3. Smooth muscle relaxation induced by forskolin to stimulate adenylyl cyclase was similar in WT, GCKO and SMC-GCKO aortae. RIA-based detection of basal cAMP levels in aortic tissue from these mice did not reveal differences. Similarly, FRET measurements showed that PGE1-induced cAMP synthesis in isolated smooth muscle cells was unaffected by NO-GC deletion. However, aortae from GCKO and SMC-GCKO animals were more sensitive to milrinone, a specific inhibitor of PDE3, as the concentration curve for relaxation was shifted to the left. Analysis of PDE3 expression in aorta showed an approximately 50% reduction in PDE3 protein and specific enzyme activity. Similar reduction was detected in platelets but not in heart tissue of GCKO animals.

Induction of SMC-specific NO-GC deletion by tamoxifen led to a parallel decline of NO-GC and PDE3 expression. Both proteins were strongly reduced already 5 days after the last tamoxifen injection. In contrast, systolic blood pressure in these animals started to rise only after 10 days and was maximal after 50 days. These data indicate that cGMP, not the increase in blood pressure, is responsible for the expression regulation of PDE3. We conclude that cGMP modulates PDE3 expression in SMC in order to keep up functional cAMP signaling.

